# Chinese Specific Characteristics of Sporadic Creutzfeldt-Jakob Disease: A Retrospective Analysis of 57 Cases

**DOI:** 10.1371/journal.pone.0058442

**Published:** 2013-03-14

**Authors:** Wei Zhao, Jia-Tang Zhang, Xiao-Wei Xing, De-Hui Huang, Cheng-Lin Tian, Wei-Quan Jia, Xu-Sheng Huang, Wei-Ping Wu, Chuan-Qiang Pu, Sen-Yang Lang, Sheng-Yuan Yu

**Affiliations:** Department of Neurology, Chinese People's Liberation Army General Hospital, Beijing, China; University of Maryland, College Park, United States of America

## Abstract

**Objective:**

Sporadic Creutzfeldt-Jakob disease (sCJD) is a fatal and transmissible neurodegenerative disorder. However, no studies have reported Chinese specific characteristics of sCJD. We aimed to identify differences in sCJD between Chinese patients and patients from other countries.

**Methods:**

The data from 57 Chinese sCJD patients were retrospectively analyzed, including demographic data, clinical manifestations, laboratory examinations, electroencephalograms (EEGs), diffusion-weighted imaging (DWI) scans, positron emission tomography (PET) scans, and pathological results.

**Result:**

The disease was pathologically confirmed in 11 patients. 39 cases were diagnosed as probable sCJD, and 7 were possible. Of the total cases, 33 were male, and 24 were female. The onset age ranged from 36 to 75 years (mean: 55.5, median: 57). Disease onset before the age of 60 occurred in 57.9% of patients. The disease duration from onset to death ranged 5–22 months (mean: 11.6, median: 11), and 51.9% of patients died 7 to 12 months after disease onset. The majority of patients presented with sub-acute onset with progressive dementia. 3 of the 9 patients who took 14-3-3 protein analysis had positive results (33.3%). The sensitivity of EEG was 79.6% (43/54). For DWI and PET examinations, the sensitivities were 94% (47/50) and 94.1% (16/17), respectively. In seven patients who did not show typical hyper-intensities on the first DWI examination, abnormalities of hypo-metabolism in the cerebral cortex were clearly detected by PET. In 13 out of the 17 patients, PET detected extra abnormal regions in addition to the hyper-intense areas observed in DWI.

**Conclusion:**

This is the first study to indicate that Chinese sCJD patients have a much earlier onset age and a longer disease duration than other populations, which is most likely related to racial differences. The longer disease duration may also be a probable characteristic of Asian populations. PET had high sensitivity for the diagnosis of sCJD.

## Introduction

Sporadic Creutzfeldt-Jakob disease (sCJD) is a fatal prion disease of the nervous system that manifests as progressive dementia, cerebellar ataxia, visual disturbance, and pyramidal and extrapyramidal tract signs, and it is caused by the accumulation of infective prion in the brain. A definitive CJD diagnosis relies on the presence of spongiform degeneration, astrocytes gliosis and nerve cell loss on autopsy or biopsy of brain tissue.

As CJD is a globally distributed disease, it has been the focus of many studies. Prior studies have reported that CJD presents differently across races. For example, the average annual age-adjusted death rate of American Indians and Alaska Natives with CJD are significantly lower than the rate for Whites and are similar to the rate for African Americans [1]. However, there have not been any reports regarding differences in sCJD between patients in China and patients in other countries. In this study, we retrospectively analyzed patient data and sought to identify specific characteristics of sCJD in Chinese patients. In addition, according to the theory that neuronal damage may lead to glucose hypo-metabolism, we attempted to determine the potential diagnostic value of PET for sCJD based on the 18F-ﬂuorodeoxyglucose positron emission tomography/CT (18F-FDG PET/CT) scans of seventeen patients.

## Materials and Methods

### Ethics Statement

The use of human clinical materials in this study was approved by the Ethical Committee of the General Hospital of the People’s Liberation Army. All of the patients and their caregivers gave written informed consent.

Fifty-seven patients with sCJD who were observed from January 1, 1992, to December 31, 2011, in the Department of Neurology, General Hospital of the People’s Liberation Army, were enrolled in this study. General Hospital of the People’s Liberation Army, which is also called Chinese PLA General Hospital or PLA 301 hospital, is one of the best hospitals in China and located in Beijing. The patients coming to this hospital are from all over the country, especially the patients with rare or complicated disease. The 57 cases in our study were from 17 provinces, across more than two-thirds area of China. The patients were diagnosed according to the updated clinical diagnostic criteria for sCJD published in 2009 [2]. All patients were without geographic-, seasonal- or occupational-related events and had no history of potential iatrogenic exposure from human cadaveric pituitary hormones, dura-mater implants, corneal grafts or neurosurgery. No patients in the study had either a family history of CJD or apparent contact with CJD patients. A diagnosis of paraneoplastic neurological syndrome was excluded for all fifty-seven patients.

The data were sourced from medical records and attending medical care providers. Follow-up information on patient condition and time of death after discharge was collected through interviews, telephone calls or comprehensive questionnaires. 14-3-3 protein was determined in western blot by standard methods in the same institution [3]. The procedure of pathological morphology observation was as follows: brain tissue with formaldehyde 24 hours, embedded in paraffin, making pathological section and HE staining. We retrospectively reviewed the data of the fifty-seven patients, including demographic data, clinical symptoms and signs, laboratory examinations, electroencephalogram (EEG), diffusion-weighted imaging (DWI), 18F-FDG PET/CT, biopsy and autopsy, focusing on the differences between patients in China and patients in other countries.

## Results

Eleven of the fifty-seven enrolled patients were pathologically confirmed to have sCJD (one case by autopsy and ten cases by biopsy). Thirty-nine cases were probable diagnoses of sCJD and seven cases were possible diagnoses.

### Clinical Data

**Figure 1:**
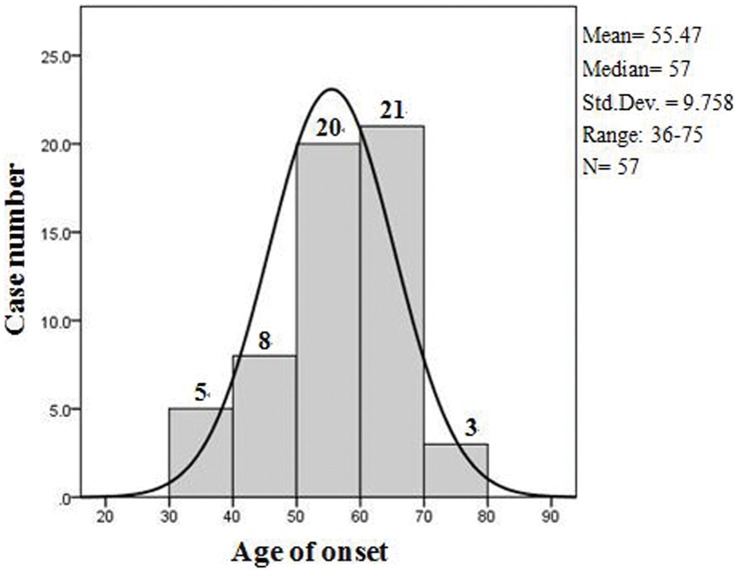
Frequency histogram of onset age with a normal curve generated by SPSS 17.0.

Thirty-three of the patients were male, and twenty-four were female (M:F = 1∶0.727). The age of onset ranged from 36 to 75 years (mean: 55.5 years, median: 57 years). As shown in Figure 1, 5 (8.8%) patients were in the 30- to 39-year-old age of onset group, 8 patients (14.0%) were in the 40–49 age of onset group, 20 patients (35.1%) were in the 50–59 age group, 21 (36.8%) were in the 60–69 age group, and the remaining 3 (5.3%) patients were in the over 70 years group. 52.6% of the patients (30/57) came to our hospital within 3 months of illness onset, and 71.9% of the patients (41/57) came to our hospital within 6 months of illness onset. The time from onset to death ranged from 5 to 22 months (mean: 11.6 months, median: 11 months). Fifty-four patients died, and one case was lost to follow up. Two patients are still alive and have lived for more than 7 months since their disease onset. The two living patients and the one lost to follow up were all diagnosed as probable sCJD according to the diagnostic criteria [2]. Among the cases in which the patients died, 51.9% of the patients (28/54) died 7 to 12 months after disease onset, and 31.5% of the patients (17/54) lived longer than 1 year after disease onset. Only 16.7% of the patients (9/54) died within 6 months of illness onset.

**Figure 2:**
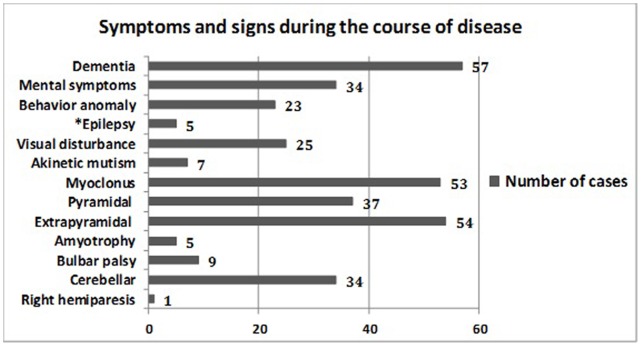
Symptoms and signs during the course of the disease. Figure 2 shows clinical manifestations observed during the course of disease and the number of cases with each manifestation. * No history of epilepsy before illness.

Twelve cases were acute onset, forty-one cases were sub-acute onset, and four cases were chronic. The predominant onset symptom was progressive dementia (50.9%, 29/57), followed by cerebellar ataxia (22.8%, 13/57), visual disturbance (17.5%, 10/57), mental disorders (5.3%, 3/57), and Parkinson syndrome (3.5%, 2/57). It is worth noting that one probable sCJD patient had an onset of right hemiparesis without any other evidence supporting a diagnosis of stroke. Patient symptoms and signs over the course of the disease are summarized in Figure 2.

### Auxiliary Examination

**Figure 3:**
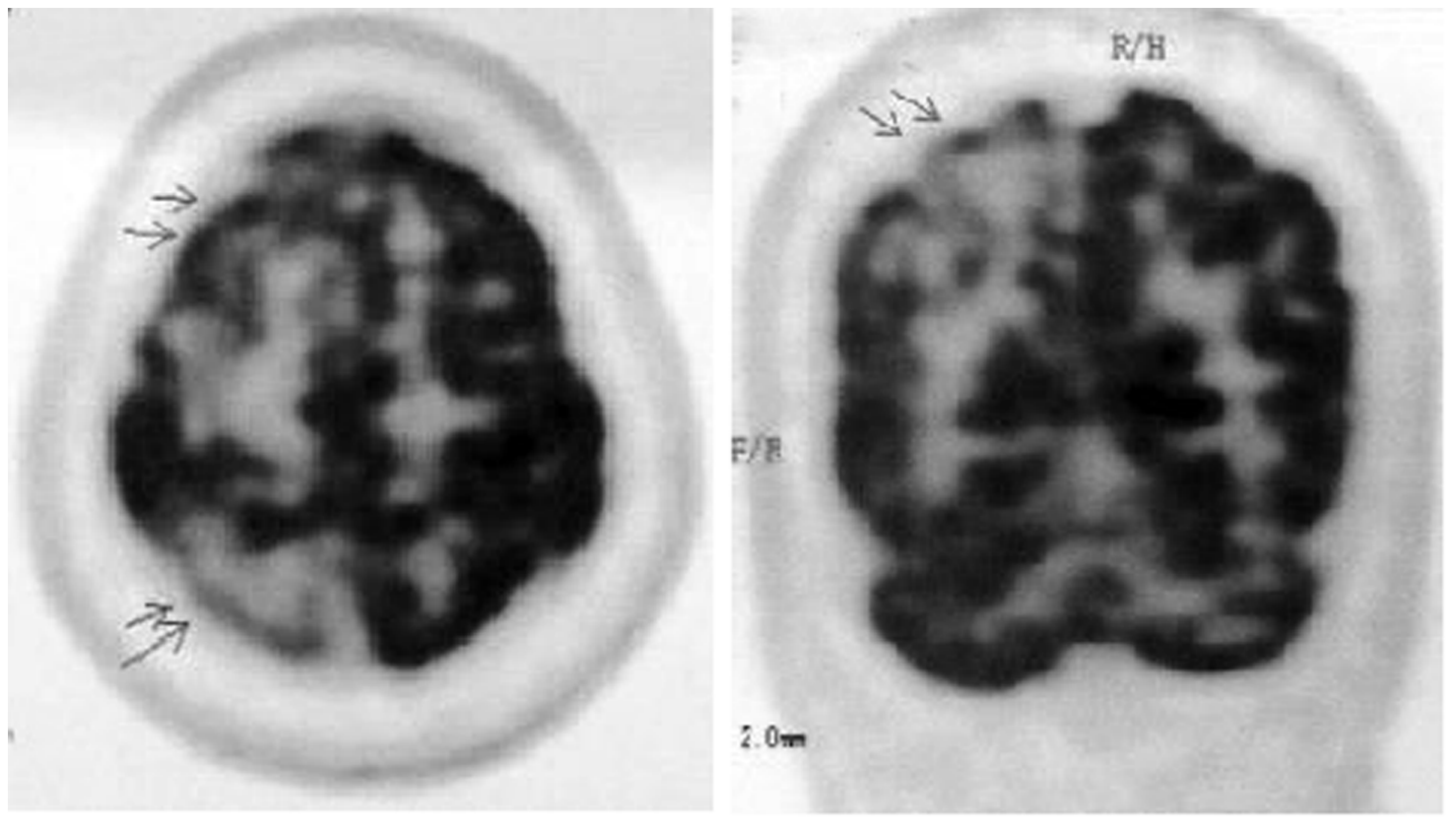
PET images of the same sCJD patient. The two images of positron emission tomography/CT scan in the same patient shows decreased ﬂuoro-2-deoxy-glucose (FDG) uptake in right frontal and parietal cortices.

In nine patients (four pathologically confirmed cases and five probable cases), 14-3-3 protein analysis of the cerebrospinal ﬂuid(CSF) showed that only three probable patients had a positive test result, with a sensitivity of 33.3%, which is much lower than other reports. The sensitivity of periodic sharp wave complexes (PSWCs) in EEG was 79.6% (43/54). Of the fifty patients who underwent DWI examination, abnormal hyper-intensity was observed in forty-seven patients (sensitivity: 94%), with 45/50 patients having abnormalities in the cortex, 25/50 in the basal ganglia and 8/50 in the thalamus. Lesions in both the basal ganglia and the cerebral cortex were found in 23/50 (46.0%) patients. Two cases only showed hyper-intensity in the basal ganglia during the course of the disease. Three possible patients had normal DWI scans. The abnormal regions may have been unilateral at disease onset but later became bilateral. The signal intensity sometimes decreased as the disease advanced. Seventeen patients underwent PET examination, and sixteen of them showed hypo-metabolism (sensitivity: 94.1%): 16/17 showed hypo-metabolism in the cortex, 11/17 in the basal ganglia, and 7/17 in the thalamus. One probable patient did not show any abnormalities on PET. In the seven patients who did not show typical hyper-intensities in the first DWI examination, abnormalities of hypo-metabolism in the cerebral cortex were clearly detected by PET at the same time of the first DWI scan. Two patients with extrapyramidal tract signs had no abnormalities in the basal ganglia using DWI, but hypo-metabolism in the basal ganglia was detected by PET. Additionally, in thirteen out of the seventeen patients who underwent PET, extra abnormal regions in addition to the hyper-intense areas on DWI were detected. The PET images of sCJD patients were shown in Figure 3.

### Pathology

**Figure 4:**
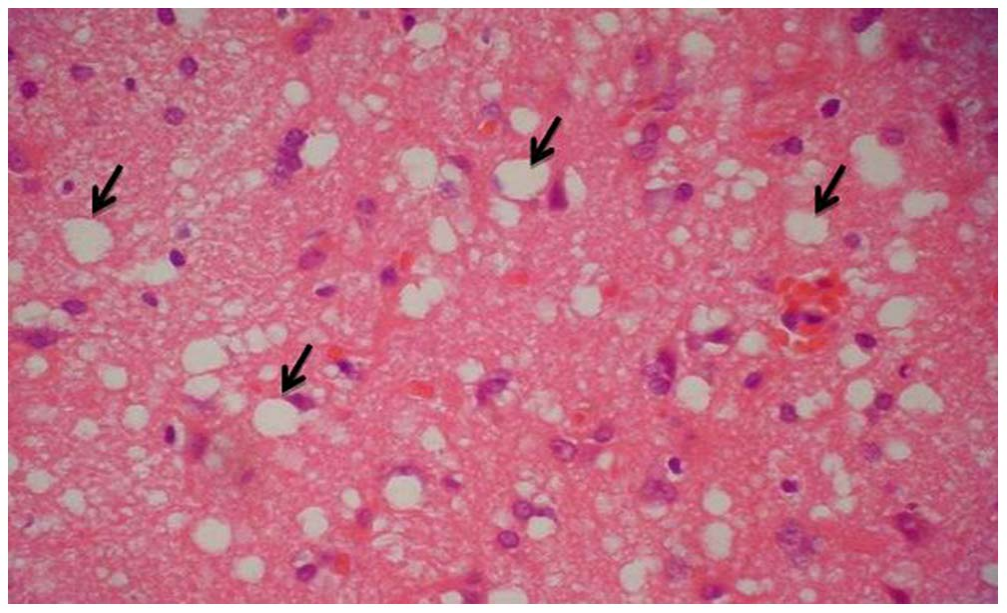
Pathologic image of sCJD. Figure 4 is a pathologic image from right frontal lobe of a biopsy case. It shows the classic pathological features of CJD: spongiform degeneration, astrocytes gliosis and nerve cell loss. The black arrows show the diffuse spongiform changes.

One case examined by autopsy and ten cases examined by biopsy presented the classic pathological features of CJD: spongiform degeneration, astrocytes gliosis and nerve cell loss. The diffuse spongiform changes are shown in Figure 4 (inset, black arrows).

## Discussion

CJD is widely recognized in the Western countries. However, the research on CJD started late in China, and the diagnostic rate of CJD is lower than in other countries. According to the search results in medical article database, the total number of CJD cases reported in China from January 1, 1981 to January 1, 2000 is less than 35. In 2007, one domestic study with seven years data reported only 23 cases [4]. And in 2008, another study in China with fifteen years data reported only 30 cases [5]. China CJD surveillance started in 2006 but the project has not been well completed. The patients’ data in surveillance program were limited, lacking detailed data of disease course and MRI result, and the surveillance members did not do follow-up observations. Among the national studies on sCJD with detailed disease history, clinical manifestations, 14-3-3 protein analysis, EEG, MRI, pathology and follow-up results, our study has the largest case number and covers the longest time span of data collection in China. In this study, we retrospectively analyzed the data of fifty-seven Chinese sCJD patients observed from January 1, 1992, to December 31, 2011, and found significant differences and similarities between Chinese patients and patients from other countries.

We compared the onset age of Chinese sCJD patients with patients from other countries. In this study, the mean age of onset in Chinese patients was 55.5 years, and the median age of onset was 57 years, ranged from 36 to 75 years. Other studies of Chinese sCJD patients showed similar results as follows. One domestic study showed that the mean onset age of 23 sCJD cases was 59.4 years [4], and another study of 30 Chinese sCJD cases reported the mean onset age was 53.3 years [5]. The report based on 2008 surveillance data in China showed that the mean age of onset in probable and possible Chinese sCJD patients were 56.7 and 57.4 years old, respectively [6]. The results of studies above were all similar to ours. In contrast, other populations had a much older onset age, including 66 years in Japan (mean) [7], [8], 64.8 years in Australia (mean) [9], 65.2 years in Germany (median) [10], and 64 years in twelve countries (median) [2]. One study also showed that the median age at death of American patients was 68 years [11]. Because most sCJD patients die within two years of disease onset, the median age of onset in America was similar to European countries but higher than China. Additionally, 57.9% of patients in our study were younger than sixty at the time of onset compared to 23.3% of patients in a study conducted by EUROCJD (the prospective CJD surveillance program conducted by the European Union and allied countries) [12]. The onset age of sCJD in China was approximately 8–9 years younger than in other countries. A younger age of disease onset is a specific feature of Chinese sCJD patients and is probably related to racial differences. Detailed information regarding onset age and disease duration is summarized in Table 1.

**Table 1:** Onset age and disease duration in different countries.

	Mean		Median	
Country	Onset age(years) (range)	Duration from onset todeath (months) (range)	Onset age(years) (range)	Duration from onset todeath (months) (range)
China (data from this study)	55.5 (36–75)	11.6 (5–22)	57 (36–75)	11 (5–22)
Japan[^7^]	66 (27–89)[^7^], [^8^]	15.7 (1–126)	NA*	NA
Australia[^9^]	64.8 (25–89)**	6.6 (1–60)	NA	NA
Germany[^10^]	NA	NA	65.2 (46.5–85.1)	7.0 (1.1–64.3)
Twelve countries*** [^2^]	NA	NA	64.0 (35.3–85)	6.4 (1.0–56.3)

* NA means “not available”, which is due to the different statistical methods. Some studies used “mean” onset age and “mean” disease duration, and others used “median”. “NA” indicates that there was no record of this type of statistic in the methods description.

** We calculated the mean onset age in Australia based on the number of cases (351), the mean age at death (65.3 years) and the mean duration (6.6 months) mentioned in the referenced report [9].

*** The twelve countries are Argentina, Belgium, Canada, France, Germany, Italy, the Netherlands, Australia, Portugal, Slovenia, Switzerland and the United Kingdom [2].

Another unique feature of Chinese sCJD patients was a longer duration from disease onset to death. The mean disease duration in our study was 11.6 months, and the median duration was 11 months, ranged from 5 to 22 months. In contrast, other studies reported 6.6 months (mean, Australia [9]), 7.0 months (median, Germany [10]) and 6.4 months (median, data from twelve countries [2]) (Table 1). In this study, only 16.7% of patients had a disease duration less than 6 months, and 31.5% of patients survived for longer than 1 year. In the EUROCJD study, 58.8% of patients died within 6 months of illness onset, and only 14.2% of patients lived longer than 1 year [12]. The disease duration of sCJD patients was longer in China, and a similar situation was also observed in Japan (mean duration, 15.7 months) [7], where the disease duration was much longer than in Western countries. These results indicated that the longer disease duration was not only a specific feature of Chinese patients but was most likely due to a difference between patients in Asian and Western countries.

Prior studies suggested a possible reason for why patients with sCJD lived longer in some countries. In Western countries, because of ethical and financial concerns, intensive life-sustaining treatments are not commonly provided to patients with progressive, fatal neurological conditions [7], [13]. In Japan, however, the social and ethical environment allows patients with end-stage disease to receive intensive life-sustaining treatments, even after deterioration to akinetic mutism [13]–[15]. In addition, a well-organized health care system reduces the financial burden on patients and their families. Thus, some researchers have suggested that these elements prolong the patients’ lives [7].

However, we disagree with this opinion. First, before coming to our department, eleven of the fifty-seven patients had endured the disease for approximately seven months without any treatment or nursing. In particular, three chronic onset patients had a one-year disease duration prior to clinical diagnosis. Additionally, due to social and ethical factors, the physicians and patients in China had a negative attitude toward using life-sustaining treatment for this rapidly progressing and fatal disease compared to the positive Japanese attitude toward treatment for end-stage patients. Furthermore, the financial burden forced some patients to terminate treatment or nursing because of limitations in the public medical insurance system in China. Even accounting for these situations, Chinese patients still had longer disease durations than patients in Western countries. Therefore, we suggest that the longer duration was not related to better care but was most likely due to racial differences. In considering the similarity in disease duration between China and Japan, there was a possibility that the longer sCJD duration was a characteristic of Asian populations. Further qualitative and quantitative research is needed in this regard.

In this study, 14-3-3 protein analysis of the cerebrospinal ﬂuid of nine patients (four pathologically confirmed cases and five probable cases) showed that only three probable patients had a positive test result, with a sensitivity of 33.3%, which was much lower than some reports of 86%–97% sensitivity [16]. Even four pathologically confirmed cases had negative results. Although some studies have suggested that analyzing 14-3-3 protein in the CSF is a reliable marker for sCJD, with a high sensitivity (86%–97%) and specificity (68%–78%) [16], others have questioned its diagnostic value and showed that only 17 of 32 definite sCJD patients (biopsy- or autopsy-confirmed diagnoses) had positive results on the 14-3-3 test, yielding a sensitivity of only 53% [17]. Due to the limited number of cases tested for 14-3-3 protein (only nine in this study), our results should only be used for reference. With respect to the detection of PSWCs in EEG, our results showed higher sensitivity (79.6%, 43/54) than other studies (44% [2], 64% [18], 66% [19]). PSWCs become obvious at around 8 to 12 weeks after the onset of clinical symptoms in most cases and occur even later in a few cases [19], [20]. We performed repeated 24-hour ambulatory electroencephalograms (24 h AEEG) on patients to capture typical PSWCs, which were detected at least four times in every patient who received the examination. Repeated examination is a probable reason for the higher sensitivity observed in this study.

Despite these differences, the onset style, clinical manifestations and DWI findings of Chinese sCJD patients were similar to those in previously published reports from other parts of the world. Most patients were sub-acute onset with the main symptom of rapidly progressive dementia followed by myoclonus, visual disturbance, and pyramidal and extrapyramidal tract signs as the disease progressed. Some departments have reported cases with stroke-like onset [21]–[23]. One such case was also observed in China. One patient in our study developed right hemiparesis without any other evidence to support a diagnosis of stroke. For the abnormal hyper-intensities observed in DWI, the sensitivity (94%) for diagnosis was similar to other reports (92.3%-96%) [24], [25].The abnormal regions may have been unilateral at the beginning of the disease but subsequently became bilateral. The signal intensity sometimes decreased as the disease advanced. This finding was reported with detailed analysis in our prior long-term follow-up study [26] and was consistent with other reports [24], [27].

In addition, we retrospectively analyzed the results of positron emission tomography (PET) with ^18^ﬂuoro-deoxyglucose in seventeen out of the fifty-seven patients. There is a theory that neuronal damage may lead to glucose hypo-metabolism. Sixteen of the seventeen patients presented with hypo-metabolism on PET, with a sensitivity of 94.1%. As one of the most sensitive modalities for observing tissue metabolism, PET can reveal reductions in cerebral glucose metabolism and has great value for earlier diagnosis of sCJD. Abnormalities can be found earlier using PET compared to DWI. Abnormalities of hypo-metabolism in the cerebral cortex were clearly detected by PET in seven patients who did not show typical hyper-intensities in the first DWI examination. Besides, in thirteen patients, PET imaging detected extra abnormal regions with hypo-metabolism in addition to the hyper-intense areas revealed by DWI. Two patients with extrapyramidal tract signs showed no abnormalities in the basal ganglia on DWI but presented hypo-metabolism in the basal ganglia on PET. In conjunction with the high sensitivity, PET has a high significance for finding early-stage tumors. The incidence of tumors in population is higher than before, and some patients with paraneoplastic syndrome have similar clinical manifestations with CJD. Based on the PET examination, two suspected sCJD patients were finally diagnosed with paraneoplastic syndrome, and early-stage tumors were found. One prior study also indicated that PET was useful to differentiate CJD from other neurodegenerative disorders [28].While the sensitivity of PET was high in our study, there was no control group analyzed to determine specificity. More research with control groups and experimental groups, such as patients with Alzheimer disease, CO poisoning and dementia with Lewy bodies, is needed.

A limitation of this study was the lower autopsy and biopsy rate of CJD cases compared with other countries. Even so, among the Chinese studies about sCJD, our study still had the high rate (19.3%, 11/57) of pathological examination. It is difficult to get the pathological samples of CJD patients in China. Most surgical and pathology departments are not willing to do autopsy or biopsy on CJD patients because CJD is infectious and fatal, and the sterilization methods for instruments that come in contact with infectivity tissues of CJD patients have not been promoted in China. According to the traditional policy in most Chinese hospitals, the instruments used in CJD patients can not be used in other patients again. Besides, due to the influence of Chinese traditional culture, most people want to keep body complete and refuse autopsy. Under this condition, we will make diagnosis in strict accordance with the clinical criteria and try to do autopsy or biopsy in suspected sCJD patients in order to make the diagnosis more exact.

In conclusion, this is the first report to detail specific disease characteristics of Chinese sCJD patients. In China, the onset age of sCJD was much earlier and the disease duration was much longer compared with other countries, and these characteristics are most likely related to racial differences. The longer disease duration was not only a special feature of Chinese patients but most likely represents a difference between patients in Asian countries and in Western countries. The sensitivity of the EEG in our study was slightly higher than observed in other studies. Regarding onset style, clinical manifestations, and DWI findings, Chinese patients were similar to other patient populations. PET has high sensitivity for the early diagnosis of sCJD.
